# Role for PKC *δ* in Fenretinide-Mediated Apoptosis in Lymphoid Leukemia Cells

**DOI:** 10.1155/2010/584657

**Published:** 2010-06-08

**Authors:** Vivian R. Ruvolo, Kul B. Karanjeet, Todd F. Schuster, Rhoderick Brown, Yibin Deng, Edward Hinchcliffe, Peter P. Ruvolo

**Affiliations:** ^1^Section of Signal Transduction and Apoptosis, Hormel Institute, University of Minnesota, Austin, MN 55912, USA; ^2^Section of Cellular Dynamics, Hormel Institute, University of Minnesota, Austin, MN 55912, USA; ^3^Section of Membrane Biochemistry, Hormel Institute, University of Minnesota, Austin, MN 55912, USA; ^4^Section of Cell Death and Cancer Genetics, Hormel Institute, University of Minnesota, Austin, MN 55912, USA; ^5^Department of Stem Cell Transplantation, MD Anderson Cancer Center, 1515 Holcombe Blvd, Houston, Tex 77030, USA

## Abstract

The synthetic Vitamin A analog fenretinide is a promising chemotherapeutic agent. In the current paper, the role of PKC *δ* was examined in fenretinide-induced apoptosis in lymphoid leukemia cells. Levels of proapoptotic cleaved PKC *δ* positively correlated with drug sensitivity. Fenretinide promoted reactive oxygen species (ROS) generation. The antioxidant Vitamin C prevented fenretinide-induced PKC *δ* cleavage and protected cells from fenretinide. Suppression of PKC *δ* expression by shRNA sensitized cells to fenretinide-induced apoptosis possibly by a mechanism involving ROS production. A previous study demonstrated that fenretinide promotes degradation of antiapoptotic MCL-1 in ALL cells via JNK. Now we have found that fenretinide-induced MCL-1 degradation may involve PKC *δ* as cleavage of the kinase correlated with loss of MCL-1 even in cells when JNK was not activated. These results suggest that PKC *δ* may play a complex role in fenretinide-induced apoptosis and may be targeted in antileukemia strategies that utilize fenretinide.

## 1. Introduction

Fenretinide (N-4-(hydroxyphenyl)-retinamide; 4-HPR) is a synthetic analog of Vitamin A that has shown promise as both a chemotherapeutic and chemopreventive agent in solid tumors and hematologic malignancies [1–6]. While fenretinide binding to retinoic acid receptors (RARs) can promote apoptosis in some cell types, the agent can induce death in a RAR-independent manner [1]. RAR-independent mechanisms of cell death likely involve the production of reactive oxygen species (ROS) and the generation of sphingolipid second messenger molecules [7–11]. While there have been numerous studies on fenretinide in recent years, the identification of diverse potential mechanisms for fenretinide antineoplastic activity suggests that the agent may function differently in different cell types [1, 11]. Fenretinide has been shown to activate JNK [6, 12], promote ROS generation [8], activate endoplasmic reticulum (ER) stress pathways [13, 14] as well as activate the intrinsic apoptotic pathway with various BCL2 family members as targets [15–17]. Recent studies have suggested that fenretinide may be an effective agent in the treatment of acute lymphoblastic leukemia (ALL) since the drug effectively kills ALL cell lines but not nonmalignant lymphoid cell types [5]. A recent study from the Reynolds group has demonstrated that fenretinide can synergize with ABT-737 to effectively kill ALL cells [18]. ABT-737 is a small molecule inhibitor of many antiapoptotic BCL2 family members (but not MCL-1) that is currently in clinical trials for a variety of cancers [19–21]. MCL-1 has been found to promote resistance to ABT-737-induced apoptosis and suppression of MCL-1 promotes sensitivity to the drug [19–21]. Fenretinide was found to promote MCL-1 degradation by a JNK-mediated mechanism and thus has promise in overcoming MCL-1 mediated chemoresistance in ALL and other leukemias [18]. 

Kim and colleagues have discovered that fenretinide can promote apoptosis via ROS activation of JNK, p38, ERK, and PKC though the potential PKC isoforms involved were not identified [6]. The protein kinase C (PKC) family is composed of at least 11 members with distinct functions and tissue distributions [22–25]. PKC isoforms are divided into three groups based on structural features: classical (cPKC), novel (nPKC), and atypical (aPKC). The cPKCs include PKC *α* and PKC *β* and require calcium and diacylglycerol (DAG) for activation [23]. The nPKCs include PKC *δ* and PKC *ε* and require DAG but not calcium for activation. The aPKCs include PKC *ζ* and require neither DAG nor calcium for activation. Both cPKC and nPKC members have been implicated in hematopoietic malignancies [24, 25]. More is known about PKC signaling in myeloid than in lymphoid cells. PKC *α* has been linked to leukemogenesis in B-cell chronic lymphocytic leukemia (B-CLL) [26]. PKC *α* promotes chemoresistance in ALL and acute myeloid leukemia (AML) cell lines [27, 28] and may be a negative prognostic factor in AML [29, 30]. PKC *β* may support B-CLL cells by CD5-mediated signaling [31]. PKC *ε* has been implicated in hairy cell leukemia as an activator of ERK and RAC1 [32]. The role of PKC *δ* in leukemia is more complicated. Unlike PKC *α*, PKC *β*, and PKC *ε* which generally regulate survival signaling pathways, PKC *δ* is regarded as a stress kinase [23, 33–36]. Cells derived from PKC *δ* null mice are resistant to apoptosis in response to chemotherapy drug or irradiation [37]. The mechanism how PKC *δ* supports apoptosis is complex. In response to a stress challenge, tyrosine phosphorylation of PKC *δ* promotes its translocation to the nucleus where it is cleaved by Caspase 3 [33–36]. The cleaved PKC *δ* is active and targets a number of nuclear substrates that may be essential for the induction of cell death including Lamin, DNA dependent Protein Kinase (DNA-PK), and p53 [33–36]. Cleaved PKC *δ* has been suggested to target MCL-1 for degradation [38]. Considering that fenretinide promotes MCL-1 degradation in ALL cell lines [18], the possibility arises that the fenretinide-induced apoptosis may involve PKC *δ*. In the present study, we examined PKC *δ* expression and cleavage and MCL-1 expression in response to fenretinide in three ALL cell lines (REH, CCRF-CEM, and MOLT4) and in a Mixed Lineage Leukemia (MLL) cell line (RS4;11). Fenretinide promoted cleavage of PKC *δ* and suppression of MCL-1 in cell lines sensitive to the drug (RS4;11 and CCRF-CEM) but not in cell lines that were more resistant (REH and MOLT4). In CCRF-CEM cells, fenretinide promoted translocation of JNK from the cytosol to the nucleus. Consistent with the findings of the Reynolds group [18], fenretinide likely promotes MCL-1 protein degradation since fenretinide did not inhibit MCL-1 gene expression in three of the four leukemia lines and the drug actually promoted gene expression in REH cells (perhaps as part of an initial SOS response for these cells). Fenretinide promoted ROS production in three of the four cell lines. The antioxidant Vitamin C protected all cell lines from the drug and suppressed PKC *δ* cleavage suggesting that the mechanism by which fenretinide promotes death involves ROS and PKC *δ*. Suppression of PKC *δ* in CCRF-CEM cells by shRNA sensitized the cells to fenretinide suggesting that loss of the kinase may also be important in the drug's mode of action. Basal ROS production was increased in cells with reduced PKC *δ* suggesting that the increase in sensitivity to the drug may be due to enhanced ROS generation. The findings in this study suggest that PKC *δ* plays an important role in fenretinide-induced apoptosis in lymphoid leukemia cell lines.

## 2. Materials and Methods

### 2.1. Cell Lines and Plasmid DNAs

HEK-293T cells were obtained from the ATCC (Manassas, VA) and maintained in DMEM supplemented with 10% fetal bovine serum at 37°C in 5% CO_2_. REH, CCRF-CEM, MOLT4, and RS4;11 cells were obtained from the ATCC and maintained in RPMI 1640 + 10% fetal bovine serum at 37°C in 5% CO_2_. A set of four shRNAs targeting PKC *δ* from the lentiviral shRNA library of The RNAi Consortium (designated TRCN0000010193, TRCN0000010194, TRCN0000010202, and TRCN0000010203) was obtained from Open Biosystems (Huntsville, AL). Lentiviral packaging plasmids MD2.G (Addgene plasmid 12259) and psPAX2 (Addgene plasmid 12260) were constructed by the laboratory of Didier Trono and obtained from Addgene (Cambridge, MA). A control lentiviral transfer plasmid, pLKO.1 TRC control (Addgene plasmid 10879), was constructed by the laboratory of David Root [39] and obtained from Addgene. 

### 2.2. Lentiviral Transduction of shRNA

Each TRC lentiviral shRNA plasmid was transiently cotransfected in an equimolar mix with the lentiviral packaging plasmids into HEK 293T cells using Lipofectamine 2000 (Invitrogen, Carlsbad, CA) as directed by the manufacturer. Lentiviral supernatants were harvested at 48 hours post transfection first by centrifugation for ten minutes at room temperature at 800 ×*g* and then by filtration through 0.45 *μ*M surfactant-free cellulose acetate to assure complete removal of producing cells. Polybrene (Chemicon, Temecula, CA) was then added to a concentration of 8 *μ*g/mL, and the resulting virus stock was used at once to spinoculate CCRF-CEM cells. CCRF-CEM cells were resuspended at a concentration of 0.5 million cells per mL of virus stock, transferred to the wells of a 12 well tissue culture cluster, and centrifuged for 45 minutes at 30°C at 1300 ×*g*. After the addition of one volume of fresh virus stock, the cells were subject to a second round of centrifugation, followed by incubation at 37°C in 5% CO_2_ for 60 minutes. The infected cells were then washed twice with growth medium to remove the polybrene and allowed to grow for two doubling times (42–44 hours), after which time they were subject to selection with 1.0 *μ*g/mL puromycin (InvivoGen, San Diego, CA). Puromycin-resistant pools of infected cells were assessed for PKC *δ* knockdown by Western analysis.

### 2.3. Analysis of ROS Generation

ROS generation assay was performed as reported by Kang and colleagues [18]. Cells were pretreated with 0.4 M Vitamin C for 2 hours prior to treatment with either vehicle (0.1% DMSO) or 1 *μ*M fenretinide for 24 hours. Cells were centrifuged and resuspended in warm (37°C) medium containing 10 *μ*M 5-(and-6)-carboxy-2′, 7′-dichlorodihydrofluorescein diacetate (carboxy-H2DCFDA; Invitrogen) and incubated for 20 minutes at 37°C. Cells were then centrifuged and washed with phosphate-buffered saline, and then analyzed on a Becton Dickinson FACSCalibur flow cytometer (BD Biosciences, San Jose, CA).

### 2.4. Analysis of Cell Death and Apoptosis by Annexin V Staining

Cells were treated with varying doses of fenretinide (Calbiochem, San Diego, CA) for 24 hours. Where appropriate, cells were pretreated 2 hours with Vitamin C (Sigma, St. Louis, MO), Caspase 8 inhibitor (Calbiochem), or Caspase 9 inhibitor (Calbiochem) or 1 hour with Bryostatin-1 (Calbiochem) prior to the addition of fenretinide. Cell viability was measured by trypan blue dye exclusion and apoptosis was evaluated using the Annexin V-FITC Apoptosis Detection Kit (MBL International, Woburn, MA). The cells were washed twice with PBS and resuspended in 450 *μ*L 1 X Annexin Binding Buffer. 5 *μ*L of Annexin V-FITC and 5 *μ*L of PI were added to each tube. The cells were mixed gently and incubated for 5 minutes in the dark at room temperature. Cells were analyzed on a Becton Dickinson FACSCalibur flow cytometer (BD Biosciences, San Jose, CA), placing the FITC signal in FL1 and the PI signal in FL2. Intact cells were gated in the FSC/SSC plot to exclude small debris. Cells in the lower right quadrant of the FL1/FL2 dot plot (labeled with Annexin V-FITC only) are considered to be in early apoptosis, and cells in the upper right quadrant (labeled with Annexin V-FITC and PI) are in late apoptosis/necrosis.

### 2.5. Western Analysis

Total protein (2 × 10^5^ cell equivalents) was subjected to SDS-PAGE using antibodies specific for the analyzed proteins. Antibodies used were Caspase 3 (Santa Cruz Biotechnology, Santa Cruz, CA), Caspase 8 (Santa Cruz Biotechnology), Caspase 9 (Santa Cruz Biotechnology), PARP (Santa Cruz Biotechnology), PKC *α* (Santa Cruz Biotechnology), PKC *β*I (Santa Cruz Biotechnology), PKC *β*II (Santa Cruz Biotechnology), PKC *δ* (Santa Cruz Biotechnology), PKC *ε* (Santa Cruz Biotechnology), p-JNK (Cell Signaling, Danvers, MA), JNK (Cell Signaling), BCL2 (Dako, Carpinteria, CA), BCL-X_*L*_ (Santa Cruz Biotechnology), MCL-1 (Santa Cruz Biotechnology), and Tubulin (Sigma).

### 2.6. Fluorescence Microscopy

Cells were suspended in RPMI media and sedimented onto round poly-L-lysine coated coverslips (Biocoat, BD Biosciences, Bedford, MA), using a Sorval RC-6 high speed centrifuge (Thermo Fischer, Pittsburgh, PA), with an HB-6 swinging bucket rotor and custom made tube inserts at 1200 × G for 15 minutes. Coverslips were fixed in −20°C methanol, and processed for immunofluorescence. Cells were immunolabeled with a monoclonal antibody against *α*-tubulin (Sigma, St. Louis MO), and a polyclonal antibody against PKC-*δ*. Secondary antibodies goat antimouse Alexa fluor 488 and goat antirabbit Alexa fluor 594, and counter stained with DAPI. Cells were imaged with a Leica DM RXA-2 microscope (Leica Microsystems, Bannockburn, IL), with fluorescence optics using a Leica 63 × 1.4 NA Apochromat CS objective and a Hamamatsu ORCA-ER cooled CCD camera (Hamamatsu, E. Bridgewater, NJ). Images were collected as a Z series of 0.5 *μ*M, and were deconvolved by constrained iterative, blind deconvolution using Simple PCI imaging software (Hamamatsu Imaging, Sewickley, PA). Z series are presented as a maximal projection and final images were cropped and adjusted for contrast in Photoshop (Adobe, Mountain View, CA).

### 2.7. Gene Expression Analysis

Total RNA was extracted from cells using TriReagent (Sigma) as directed by the manufacturer. To ensure complete removal of trace genomic DNA or other factors that could interfere with downstream enzymatic processes, all RNA samples were subjected to final purification using RNeasy Mini Columns (Qiagen, Valencia, CA) with on-column treatment by DNAse I as directed by the manufacturer. cDNA was prepared from 1.0 *μ*g of total RNA per 20 *μ*L mix containing 0.07 *μ*g/*μ*L random-sequence hexamer primers, 1 mM dNTPs, 5 mM DTT, 0.2 u/*μ*L SuperAsin RNAse inhibitor (Ambion, Austin, TX), and 10 u/*μ*L SuperScript III reverse transcriptase (Invitrogen). RNA and primers were denatured 5 minutes at 70°C and then chilled on ice. All components except enzyme were added and the mixture was incubated at room temperature for 2 minutes to allow nucleic acids to anneal. After addition of reverse transcriptase, the mixture was incubated for ten minutes at 25°C, then one hour at 50°C, followed by heat-inactivation of the enzyme for 15 minutes at 72°C. All cDNAs were stored at −80°C when not in use. To verify the complete removal of any residual genomic material in the real-time PCR assays, we incubated in parallel 1.0 *μ*g of total RNA per 20 *μ*L of a mix containing all components except reverse transcriptase.

Real-time PCR was carried out using an ABI Model 7500 Sequence Detection System (Applied Biosystems, Foster City, CA). Duplicate 25 *μ*L reactions containing the cDNA equivalent of 50 pg total RNA were run and repeated if the Ct values were more than 0.25 cycles apart. As primers and probes we used the following TaqMan Gene Expression Assays (Applied Biosystems) as directed by the manufacturer: MCL1 assay ID Hs00172036_m1, BCL2 assay ID Hs00236808_s1, B2M assay ID Hs99999907_m1, and 18SrRNA Hs99999901_s1. We used 18S rRNA to normalize gene expression. To calculate the relative abundance (RA) of each transcript of interest relative to that of 18S, the following formula was employed: RA = 1000000 × 2^[−ΔCt]^  , where ΔCt is the mean Ct of the transcript of interest minus the mean Ct of the transcript for 18S. 

### 2.8. Statistics

Statistical analysis was performed using standard *t*-test analysis with Sigma Stat computer software (SSPS, Chicago, IL). Results are expressed as means ± SD of 3 separate replicate experiments. Values of *P* < .05 were considered significant.

## 3. Results

### 3.1. Fenretinide Promotes Cell Death in Leukemia Cell Lines by a Mechanism Involving ROS

**Figure 1:**
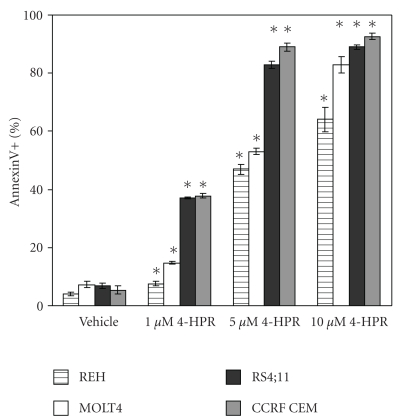
Fenretinide promotes apoptosis in leukemia cell lines. Apoptosis of human leukemia derived REH, MOLT4, RS4;11 and CCRF-CEM cells treated with vehicle (0.1% DMSO) or fenretinide (4-HPR at 1 *μ*M, 5 *μ*M or 10 *μ*M dose) for 24 hours was examined using FACSCAN analysis of Annexin V stained cells. Error bars represent the mean ± S.D. from three separate experiments. Statistically significant differences from cell viability in untreated cells (standard *t*-test; *P* < .05) are marked by “∗”.

Sensitivity to fenretinide was investigated in the T-ALL derived cell lines CCRF-CEM and MOLT4 cells, in the pre-B ALL derived cell line REH, and in the MLL derived cell line RS4;11. Cells were treated with vehicle (0.1% DMSO) or with *1* *μ*M, 5 *μ*M, or 10 *μ*M fenretinide for 24 hours and programmed cell death was assessed by Annexin V staining using FACSCAN. As shown in Figure 1, CCRF-CEM and RS4;11 cells were the most sensitive to fenretinide. At 1 *μ*M fenretinide, >30% of CCRF-CEM and RS4;11 cells were positive for Annexin V staining while higher doses of the drug induce apoptosis in the majority of cells from both cell lines (i.e., >80% at 5 *μ*M and >89% at 10 *μ*M; Figure 1). Compared to CCRF-CEM and RS4;11 cells, MOLT 4 cells were more resistant to fenretinide. As shown in Figure 1, REH cells displayed the greatest resistance to fenretinide-induced apoptosis. 

**Figure 2:**
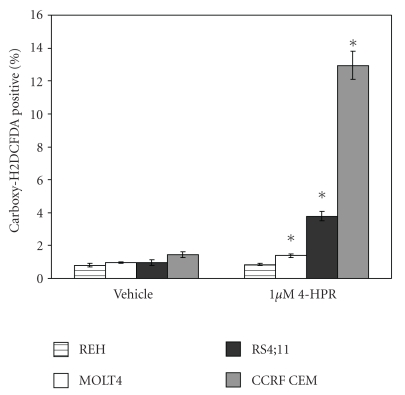
Fenretinide promotes ROS generation in leukemia cell lines. Generation of ROS in human leukemia derived REH, MOLT4, RS4;11 and CCRF-CEM cells treated with vehicle (0.1% DMSO) or fenretinide (1 *μ*M 4-HPR) for 24 hours was examined using FACSCAN analysis of Carboxy-H2DCFDA stained cells. Error bars represent the mean ± S.D. from three separate experiments. Statistically significant differences from cell viability in untreated cells (standard *t*-test; *P* < .05) are marked by “∗”.

Since RS4;11 cells are p53+ and CCRF-CEM cells contain mutant p53, p53 does not appear to be required for fenretinide-induced cell death. This finding is consistent with published reports indicating that fenretinide acts to kill cells via a p53 independent mechanism [1]. Fenretinide has been shown to promote cell death via a ROS-mediated mechanism. As shown in Figure 2, treatment of cells with 1 *μ*M fenretinide for 24 hours promoted potent ROS generation in RS4;11 cells (i.e., >3.8 fold increase, *P* < .001) and CCRF-CEM cells (i.e., ~9 fold increase, *P* < .001). MOLT4 cells treated similarly with fenretinide exhibited a slight but statistically significant increase in ROS produced (i.e., 45%, *P* < .003). Treatment of REH cells with 1 *μ*M fenretinide for 24 hours had no effect on ROS generation. However, REH cells treated with 5 *μ*M drug did show a >-4 fold increase in ROS production (*P* < .002) after 24 hours (data not shown). These results suggest that there is a positive correlation between ROS generation and sensitivity to fenretinide. 

**Figure 3:**
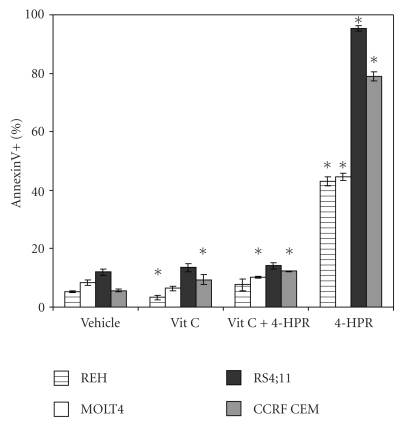
The antioxidant Vitamin C protects leukemia cell lines from fenretinide-induced apoptosis. Apoptosis of human leukemia derived REH, MOLT4, RS4;11 and CCRF-CEM cells treated with vehicle (0.2% DMSO) or fenretinide (10 *μ*M 4-HPR) for 24 hours was examined using FACSCAN analysis of Annexin V stained cells. Where appropriate, cells were pretreated for 2 hours with 0.4 M Vitamin C (Vit C). Error bars represent the mean ± S.D. from three separate experiments. Statistically significant differences from cell viability in untreated cells (standard *t*-test; *P* < .05) are marked by “∗”.

If ROS generation is critical to fenretinide-induced apoptosis, it would be expected that an antioxidant such as Vitamin C (L-abscorbic acid) would protect cells from the cytotoxic effects of the drug. REH, MOLT4, RS4;11, and CCRF-CEM cells were treated with vehicle (0.2% DMSO), 0.4 M Vitamin C, 5 *μ*M fenretinide, or a combination of Vitamin C and drug at the specified concentrations for 24 hours. Induction of apoptosis was then measured by FACSCAN analysis of cells to identify Annexin V positive cells. Vitamin C alone had no effect on RS4;11 or MOLT4 cells but did have a slight effect on the other two cells lines (Figure 3). Vitamin C promoted an increase of apoptotic CCRF-CEM cells compared to cells treated with vehicle (i.e., 9.4% versus 5.7%, resp.; *P* < .002). In REH cells, Vitamin C alone reduced the population of apoptotic CCRF-CEM cells compared to cells treated with vehicle (i.e., 5.3% versus 3.2%, resp.; *P* < .002). As shown in Figure 3, Vitamin C protected all four cell lines from fenretinide-induced apoptosis. This result strongly suggests that ROS production is a key element in the mechanism by which fenretinide kills leukemia cells. 

### 3.2. Fenretinide Promotes Caspase Activation

**Figure 4:**
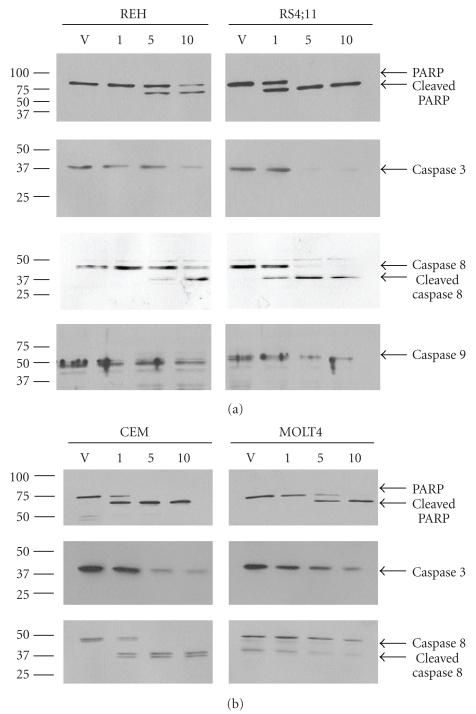
Fenretinide promotes Caspase activity in leukemia cell lines. Western blot analysis was performed using antibody against PARP, Caspase 3, Caspase 8, and Caspase 9 on total lysate (0.25 × 10^6^ cell equivalents) from REH, RS4;11, MOLT4, and CCRF-CEM (CEM) cells treated with vehicle (lane marked V; 0.1% DMSO), 1 *μ*M fenretinide (lane marked 1), 5 *μ*M fenretinide (lane marked 5), or 10 *μ*M fenretinide (lane marked 10).

**Figure 5:**
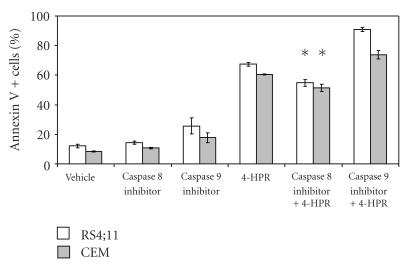
Caspase Inhibitors do not protect leukemia cell lines from fenretinide-induced apoptosis. Apoptosis of human leukemia derived RS4;11 and CCRF-CEM (CEM) cells treated with vehicle (0.2% DMSO) or fenretinide (10 *μ*M 4-HPR) for 24 hours was examined using FACSCAN analysis of Annexin V stained cells. Where appropriate, cells were pretreated for 2 hours with 20 *μ*M Caspase 8 inhibitor or 20 *μ*M Caspase 9 inhibitor. Error bars represent the mean ± S.D. from three separate experiments. Statistically significant differences from cell viability in untreated cells (standard *t*-test; *P* < .05) are marked by “∗”.

It has been previously established that fenretinide can promote apoptosis in leukemia cell lines such as CCRF-CEM by induction of mitochondrial-mediated oxidative stress [7, 8, 18]. However, a recent study has suggested that Caspase activity is not required for fenretinide-induced cell death in lymphoid leukemia cell lines [18]. Caspase activation in response to fenretinide was determined in the lymphoid leukemia cell lines. Cells were treated with varying doses of drug for 24 hours and Caspase activation was observed by Western analysis of PARP cleavage. As shown in Figure 4, PARP cleavage was observed in RS4;11 and CCRF-CEM cells treated with 1 *μ*M fenretinide while complete PARP cleavage is seen with 5 *μ*M of the drug. REH cells and MOLT4 cells, which are relatively more resistant to fenretinide-induced cell death compared to RS4;11 and CCRF-CEM cells, displayed less PARP cleavage in response to fenretinide. In fact, PARP is only partially cleaved in REH cells even when treated with 10 *μ*M fenretinide (Figure 4). RS4;11 cells display the greatest activation of executioner Caspase 3 (as observed by loss of pro-Caspase) with fenretinide treatment. Interestingly, activation of Caspase 9 (which mediates the intrinsic/mitochondrial apoptotic pathway) was not very pronounced in RS4;11 or REH cells and was not detected in CCRF-CEM or MOLT4 cells (data not shown). As shown in Figure 4, fenretinide activated Caspase 8 in the RS4;11 and CCRF-CEM cell lines (as observed by cleavage of the Caspase) but not in MOLT4 cells. There was limited activation of Caspase 8 in REH cells compared to RS4;11 and CCRF-CEM cells. A dose of 10 *μ*M fenretinide is required to observe some Caspase 8 cleavage in REH cells while 5 *μ*M drug results in near complete cleavage of the Caspase in both RS4;11 and CCRF-CEM cells (Figure 4). However, fenretinide-induced apoptosis in RS4;11 or CCRF-CEM cells does not require either Caspase 8 or Caspase 9. As shown in Figure 5, inhibition of Caspase 9 actually increased sensitivity to fenretinide in both cell lines. While inhibition of Caspase 8 did result in statistically significant protection from fenretinide in RS4;11 and CCRF-CEM cells (*P* = .001 and *P* = .003, resp.), the protection accorded was only partial indicating that Caspase 8 may be necessary but not sufficient for fenretinide-induced apoptosis in these cell lines. These results are similar to findings reported recently from the Reynolds group that demonstrated that fenretinide-induced apoptosis in the T-ALL-derived cell line COG-LL-317 involves a Caspase independent mechanism [18]. 

### 3.3. Fenretinide Induces Cleavage and Nuclear Translocation of PKC *δ* and Suppression of MCL-1 Expression in Leukemia Cells that Are Sensitive to the Drug

**Figure 6:**
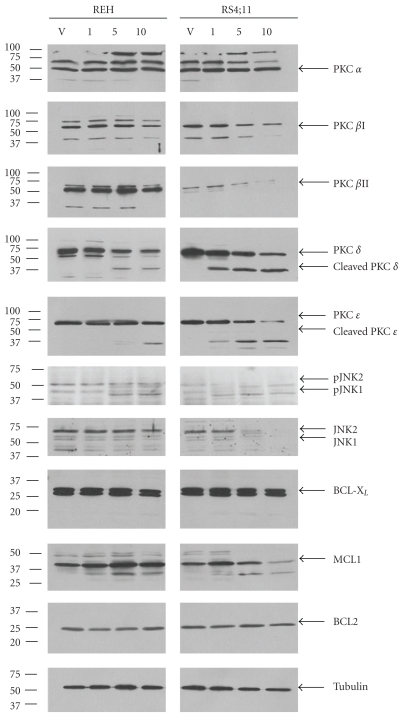
Fenretinide promotes PKC *δ* cleavage and suppresses MCL-1 expression in RS4;11 cells. Western blot analysis was performed using antibody against PKC *α*, PKC *β*I, PKC *β*II, PKC *δ*, PKC *ε*, p-JNK, JNK, BCL-X_*L*_, MCL-1, BCL2, and Tubulin on total lysate (0.25 × 10^6^ cell equivalents) from REH and RS4;11 cells treated with vehicle (lane marked V; 0.1% DMSO), 1 *μ*M fenretinide (lane marked 1), 5 *μ*M fenretinide (lane marked 5), or 10 *μ*M fenretinide (lane marked 10) for 24 hours.

**Figure 7:**
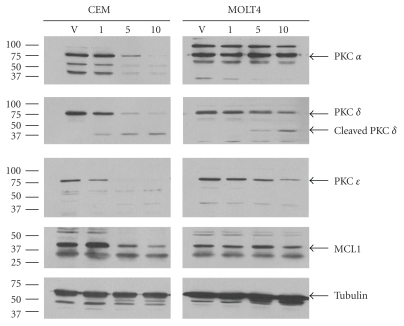
Fenretinide promotes PKC *δ* cleavage and suppresses MCL-1 expression in CCRF-CEM cells. Western blot analysis was performed using antibody against PKC *α*, PKC *δ*, PKC *ε*, MCL-1, and Tubulin on total lysate (0.25 × 10^6^ cell equivalents) from CCRF-CEM (CEM) cells and RS4;11 cells treated with vehicle (lane marked V; 0.1% DMSO), 1 *μ*M fenretinide (lane marked 1), 5 *μ*M fenretinide (lane marked 5), or 10 *μ*M fenretinide (lane marked 10) for 24 hours.

**Figure 8:**
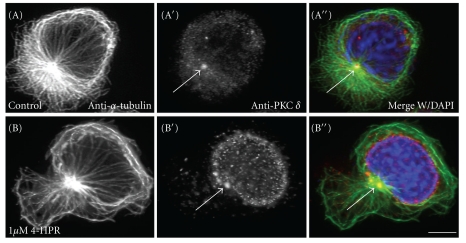
PKC-*δ* translocates into the nucleus in response to fenretinide (4-HPR) treatment. CCRF-CEM cells were treated with either vehicle (A-A′′) or 1 *μ*M 4-HPR (B-B′′) for 24 hours and then sedimented onto poly L-lysine coated coverslips. Cells were stained with anti *α* tubulin to label the microtubule network (A-B), and anti-PKC *δ* (A′-B′). In the untreated cells, the PKC *δ* localizes to the cytoplasm and is concentrated at the centrosome (A′, arrow). In the treated cells, the PKC *δ* is concentrated in the nucleus, and also at the cytoplasm (B′, arrow). Fluorescence optics Bar = 5 *μ*M.

A number of signaling pathways that may be activated by fenretinide have been identified (e.g., JNK) [6, 8, 12–14]; however, a role for PKC in fenretinide-induced apoptosis has yet to be established. A role for PKC in fenretinide-induced apoptosis in head and neck squamous carcinoma cells has been suggested but an analysis of which PKC isoforms might participate was not performed [6]. REH and RS4;11 cells were treated with varying doses of fenretinide for 24 hours and then lysed for Western blot analysis. As shown in Figure 6, fenretinide had little effect on the classical PKC isoforms (i.e., PKC *α*, PKC *β*I, and PKC *β*II) in REH cells but the expression of each of these was suppressed in RS4;11 cells. Fenretinide potently induced cleavage of novel PKC isoforms PKC *δ* and PKC *ε* in RS4;11 cells and to a lesser extent in REH cells (Figure 4). While the significance of PKC *ε* cleavage is not clear, cleavage of PKC *δ* has been implicated as a positive regulator of apoptosis [33–36]. CCRF-CEM and MOLT4 cells were similarly treated with fenretinide and expression of PKC *α*, PKC *δ*, and PKC *ε* was examined (Figure 7). As was the case for RS4;11 cells, fenretinide suppressed PKC *α* expression and promoted cleavage of PKC *δ* in CCRF-CEM cells. While the drug did suppress expression of PKC *ε*, no cleavage product was detected (Figure 5). As was the case for REH cells, fenretinide treatment of MOLT4 cells had little effect on PKC *α* expression and only promoted PKC *δ* cleavage with higher concentrations. Considering that cleaved PKC *δ* has been implicated as a positive regulator of apoptosis, it is not surprising to find that the leukemia cells that are more sensitive to fenretinide (i.e., RS4;11 and CCRF-CEM cells) exhibit greater production of cleaved PKC *δ* compared to the leukemia cells that are more resistant (i.e., REH and MOLT4). Nuclear translocation of PKC *δ* has been shown to be an important event in apoptotic stress signaling pathways involving the kinase [33–36]. To determine if fenretinide promoted nuclear translocation of PKC *δ*, subcellular localization of the kinase was examined by immunofluorescence microscopy in vehicle (0.1% DMSO) treated CCRF-CEM cells and cells treated with 1 *μ*M fenretinide for 24 hours. As shown in Figure 8, PKC *δ* is mainly found in the cytoplasm and in association with centrosomes in cells treated with vehicle. However, in cells treated with fenretinide, antibody against PKC *δ* labels the nuclear region, and is not present in the cytoplasm (Figure 8). PKC *δ* continues to localize to the centrosome in cells treated with fenretinide (Figure 8, arrow). The promotion of PKC *δ* localization to the nucleus is consistent with proapoptotic signaling described by others [33–36].

A recent study from the Reynolds group suggests that fenretinide suppresses expression of the antiapoptotic BCL2 family member MCL-1 in ALL cells [18]. Cleaved PKC *δ* has been suggested to regulate the degradation of MCL-1 during apoptosis in U.V. irradiated keratinocytes [38]. It is not surprising that fenretinide sensitive cell lines RS4;11 and CCRF-CEM display reduced expression of MCL-1 with concomitant cleavage of PKC *δ* when treated with higher doses of the drug (Figures 6 and 7). Fenretinide had no effect on the protein expression of the other antiapoptotic BCL2 family members expressed in the REH and RS4;11 cells (i.e., BCL2 and BCL-XL; Figure 6). In the Reynolds group study, JNK was implicated as regulating MCL-1 degradation by observations made using CCRF-CEM cells [18]. As shown in Figure 6, RS4;11 cells exhibit suppression of MCL-1 protein expression in response to fenretinide but do not display significant fenretinide-induced activation of JNK (as indicated by phosphorylation of the kinase). In fact, both JNK1 and JNK2 appear to be downregulated by fenretinide in RS4;11 cells at higher concentrations (Figure 6). Thus at least in RS4;11 cells, suppression of MCL-1 expression is independent of JNK. 

### 3.4. Fenretinide Regulates MCL-1 and BCL2 Gene Transcription in the Leukemia Cells

**Figure 9:**
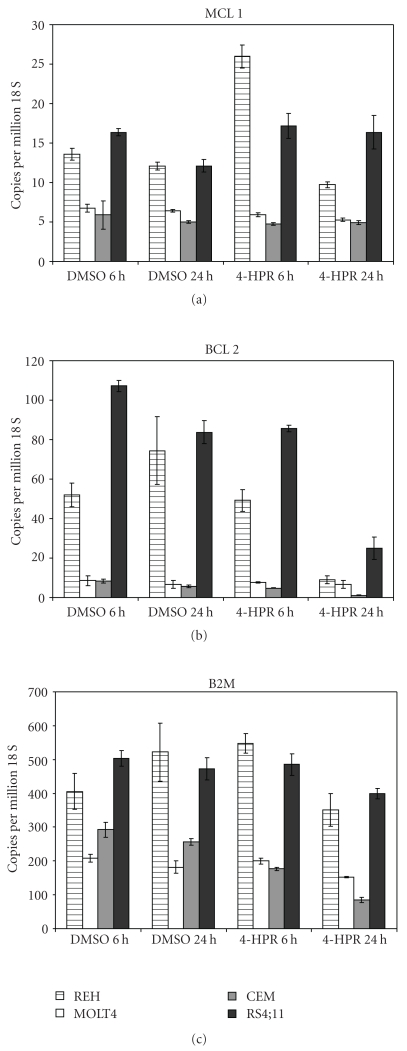
Fenretinide does not inhibit transcription of MCL-1 in leukemia cells. Real-Time-PCR was performed using cDNA derived from cells treated with vehicle (0.1% DMSO) or cells treated with 10 *μ*M fenretinide (4-HPR) for 6 hours or 24 hours. Expression of MCL-1, BCL2, and B2M genes are presented as relative to 10^6^ copies of 18S RNA.

To examine if suppression of MCL-1 expression by fenretinide might be mediated by a transcriptional mechanism, gene expression of MCL-1 as well as fellow antiapoptotic family member BCL2 was assessed in cells treated with the drug. REH, MOLT4, CCRF-CEM, and RS4;11 cells were treated with vehicle (0.1% DMSO) or 10 *μ*M fenretinide for 6 hours or 24 hours. RNA was isolated and transcription of MCL-1 and BCL2 was measured by real-time PCR (RT-PCR). Expression of 18S RNA was also measured so expression of each gene is reported as transcript per million copies of 18S. To control the effect of fenretinide on transcription overall, B2M was also measured by RT-PCR. Fenretinide does appear to have differential effects on transcription overall among the various cell lines as determined by the changes in the level of B2M. There was a slight reduction of B2M transcript observed in MOLT4 and RS4;11 cells treated with fenretinide for 24 hours (~15% reduction each; Figure 9). However, fenretinide suppressed B2M transcription by nearly 2-fold after 24 hours in REH cells and by nearly 3-fold after 24 hours in CCRF-CEM cells. As shown in Figure 9, fenretinide did not suppress MCL-1 transcription by more than 20% in any of the cells after 24 hours. In fact, there was a slight (i.e., ~35%) increase in MCL-1 transcript in RS4;11 cells when treated with the drug for 24 hours. Thus loss of transcript cannot account for the suppression of MCL-1 protein by 10 *μ*M fenretinide in these cells that was demonstrated in Figure 6. CCRF-CEM cells, which also exhibit reduced MCL-1 protein in response to the drug (see Figure 7), likewise do not exhibit much decrease (i.e., ~18%) in MCL-1 transcription. Actually relative to B2M transcription, there is a >2-fold increase in MCL-1 transcript in the fenretinide treated CCRF-CEM cells when compared to control. Fenretinide actually promoted MCL-1 protein expression in the REH cells (Figure 6). Interestingly, there was a >2-fold increase in MCL-1 gene expression in REH cells treated with 10 *μ*M fenretinide for 6 hours compared to vehicle treated control cells (Figure 9). It remains to be determined if fenretinide promotes MCL-1 protein expression in REH cells by a transcriptional mechanism. Also, it is possible that fenretinide regulates MCL-1 gene expression by other means such as a mechanism involving a micro-RNA. Still, the data presented here suggest that downregulation of MCL-1 in the fenretinide sensitive cell lines (i.e., RS4;11 and CCRF-CEM) does not occur via a transcriptional mechanism. 

Interestingly, REH and RS4;11 cells treated with 10 *μ*M fenretinide for 24 hours exhibited >3-fold reduction and >8-fold reduction, respectively, in BCL2 gene expression compared to vehicle control cells treated for 24 hours (Figure 9). Fenretinide induced loss of PKC *ε* in these cells (Figures 6 and 7). PKC *ε* has been implicated as a regulator of BCL2 gene expression in hematopoietic cells [40]. Thus fenretinide may regulate BCL2 gene expression via a PKC *ε*-mediated mechanism. It should be noted that fenretinide did not affect BCL2 protein levels after 24 hours in either REH or RS4;11; however, BCL2 protein has a relatively long half life compared to MCL-1 [41]. Overexpression of exogenous BCL2 in CCRF-CEM cells has been shown to protect the cells from the cytotoxic effects of fenretinide [17]. The effect of fenretinide to suppress BCL2 gene expression thus may be key to the cell death mechanism as the antiapoptotic protein interferes with fenretinide drug action when BCL2 gene expression is regulated by an artificial promoter (i.e., CMV promoter) [17] and thus not subject to regulation by the drug. While this potential mechanism needs to be investigated further, the ability of fenretinide to suppress BCL2 expression would have positive benefits in antileukemia strategies that would utilize fenretinide.

### 3.5. The Antioxidant Vitamin C Prevents PKC *δ* Cleavage Induced by Fenretinide

**Figure 10:**
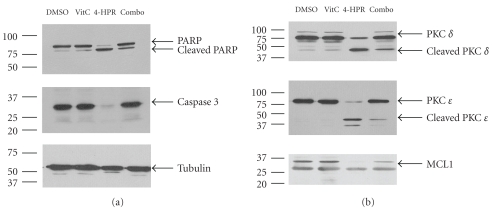
The antioxidant Vitamin C blocks fenretinide-induced cleavage of PKC *δ* in RS4;11 cells. Western blot analysis was performed using antibody against PKC *α*, PKC *β*I, PKC *β*II, PKC *δ*, PKC *ε*, p-JNK, JNK, BCL-X_*L*_, MCL-1, BCL2, and Tubulin on total lysate (0.25 × 10^6^ cell equivalents) from RS4;11 cells treated with vehicle (0.2% DMSO), 0.4 M Vitamin C (Vit C), 10 *μ*M fenretinide (4-HPR), or 10 *μ*M fenretinide after a 2 hour pretreatment with 0.4 M Vitamin C (Combo) for 24 hours.

A plausible mechanism how fenretinide promotes PKC *δ* cleavage might involve ROS activation of Caspase 3 since the protease mediates cleavage of this PKC isoform [33–36]. Nuclear translocation of PKC *δ* precedes Caspase 3 cleavage and indeed fenretinide promotes PKC *δ* nuclear translocation (Figure 8). As shown in Figure 4, Caspase 3 is potently activated and PARP cleaved in RS4;11 and CCRF-CEM cells treated with fenretinide even when cells are treated with 1 *μ*M drug. Vitamin C was shown to effectively protect the leukemia cells from fenretinide-induced apoptosis likely via a mechanism that suppresses ROS (Figure 3). If PKC *δ* participates in the death process, it would be predicted that Vitamin C would prevent fenretinide-induced cleavage of PKC *δ*. RS4;11 cells were treated with vehicle (0.1% DMSO), 0.4 M Vitamin C, 5 *μ*M fenretinide, or a combination of Vitamin C and drug at the specified concentrations for 24 hours. Protein expression was analyzed by Western analysis. Consistent with the protective effects of Vitamin C against drug-induced apoptosis, Vitamin C blocked fenretinide-induced activation of Caspase 3 (i.e., inhibited pro-Caspase cleavage) and suppressed cleavage of PARP in the RS4;11 cells (Figure 10). As shown in Figure 10, the antioxidant was effective at preventing PKC *δ* cleavage in cells treated with fenretinide. This result supports a mechanism where fenretinide promotes ROS formation to activate Caspase 3 resulting in PKC *δ* cleavage.

### 3.6. In REH Cells, the PKC Agonist Bryostatin-1 Potentiates Fenretinide-Induced Apoptosis and Suppresses PKC *δ* Expression

**Figure 11:**
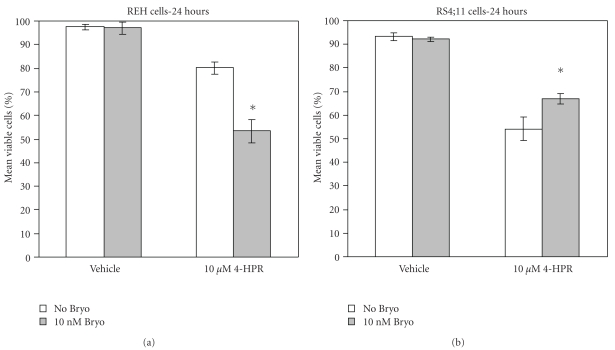
Bryostatin-1 protects RS4;11 but not REH cells from fenretinide-induced cell death. Cell death of human leukemia derived REH cells (a) and RS4;11 cells (b) treated with vehicle (0.2% DMSO) or fenretinide (10 *μ*M 4-HPR) for 24 hours was examined by trypan blue dye exclusion assay. Where appropriate, cells were pretreated for 2 hours with 10 nM Bryostatin-1 (Bryo). Error bars represent the mean ± S.D. from three separate experiments. Statistically significant differences from cell viability in untreated cells (standard *t*-test; *P* < .05) are marked by “∗”.

It has been previously demonstrated that the PKC agonist Bryostatin-1 protects REH cells from apoptosis induced by a number of clinically relevant chemotherapeutic drugs including etoposide, ara C, and adriamycin [27]. The mechanism how Bryostatin-1 protects REH cells from chemotherapeutic drugs involves, at least in part, the activation of PKC *α* resulting in the phosphorylation of BCL2 [27]. Bryostatin-1 mimics diacylglycerol (DAG) and binds to the DAG binding site of PKC resulting in activation; however, prolonged exposure to the drug can result in downregulation of particular PKC isoforms likely due to feedback mechanisms [42]. REH cells were treated with vehicle (0.2% DMSO) or 10 *μ*M fenretinide for 24 hours. Where appropriate, cells were pretreated with 10 nM Bryostatin-1 1 hour prior to addition of vehicle or fenretinide. Cell viability was determined by trypan blue dye exclusion dye assay. As shown in Figure 11(a), 10 *μ*M fenretinide kills roughly 20% of REH cells. While 10 nM Bryostatin-1 alone has no toxic effect on the REH cells, nearly half of cells treated with both 10 nM Bryostatin-1 and 10 *μ*M fenretinide were killed after 24 hours (Figure 11(a)). The increase in fenretinide-induced cell death in the REH cells due to Bryostatin-1 was statistically significant (*P* = .0006). Thus while Bryoststain-1 protects REH cells from chemotherapeutic drugs, the PKC agonist promotes cell death in response to fenretinide. To determine if Bryostatin-1 acted similarly on RS4;11 cells treated with fenretinide, RS4;11 cells were treated with fenretinide in the absence or presence of 10 nM Bryostatin-1. Since RS4;11 cells are much more sensitive to fenretinide compared to REH cells (see Figure 1), RS4;11 cells were treated with 1 *μ*M fenretinide. Bryoststin-1 actually protected RS4;11 cells from fenretinide-induced apoptosis. As shown in Figure 11(b), RS4;11 cells pretreated with 10 nM Bryostatin-1 demonstrated a significant decrease in response to 1 *μ*M fenretinide after 24 hours compared to cells treated with fenretinide alone (46% cell death versus 33% cell death resp.; *P *= .02). Thus unlike REH cells, Bryostatin-1 can protect RS4;11 cells from the cytotoxic effects of fenretinide.

**Figure 12:**
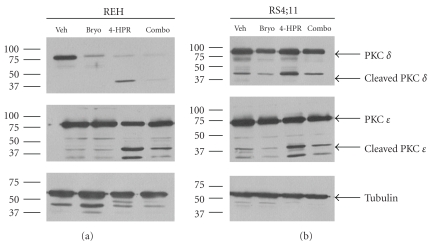
Bryostatin-1 suppresses PKC *δ* expression in REH cells. Western blot analysis was performed using antibody against PKC *δ*, PKC *ε*, and Tubulin on total lysate (0.25 × 10^6^ cell equivalents) from REH cells and RS4;11 cells treated with vehicle (0.2% DMSO), 10 nM Bryostatin-1 (Bryo), fenretinide 4-HPR), or fenretinide after a 2 hour pretreatment with 10 nM Bryostatin-1 (Combo) for 24 hours. Due to differences in sensitivity to the drug, 10 *μ*M fenretinide was used for REH cells and 1 *μ*M fenretinide was used for RS4;11 cells.

 Differential effects of Bryostatin-1 on PKC *δ* in REH cells and RS4;11 cells may provide a possible explanation for the difference in effect of Bryostatin-1 on fenretinide-induced apoptosis in the two cell lines. REH and RS4;11 cells were treated with vehicle (0.2% DMSO), 10 nM Bryostatin-1, fenretinide, or a combination of Bryostatin-1 and drug at the specified concentrations for 24 hours. REH cells were treated with 10 *μ*M fenretinide and RS4;11 cells were treated with 1 *μ*M fenretinide. Protein expression was analyzed by Western analysis. Consistent with the protective effects of Bryostatin-1 against fenretinide-induced apoptosis in the RS4;11 cells, the PKC agonist blocked fenretinide-induced cleavage of both PKC *δ* and PKC *ε* (Figure 12). Bryostatin-1 alone had no effect on PKC *ε* in REH cells though it did prevent fenretinide-induced cleavage of PKC *ε* (Figure 12). Since Bryostatin-1 augments fenretinide-induced cell death but blocks cleavage of PKC *ε* in REH cells, it is likely that cleaved PKC *ε* is not critical in the death process. Meanwhile, Bryostatin-1 potently suppressed PKC *δ* expression in REH cells and there was a near complete inhibition of PKC *δ* in REH cells treated with the combination of Bryostatin-1 and fenretinide (Figure 12). This result suggests that loss of PKC *δ* sensitizes REH cells to fenretinide-induced cell death.

### 3.7. Suppression of PKC *δ* Expression Promotes Fenretinide-Induced Apoptosis in RS4;11 and CCRF-CEM Cells

**Figure 13:**
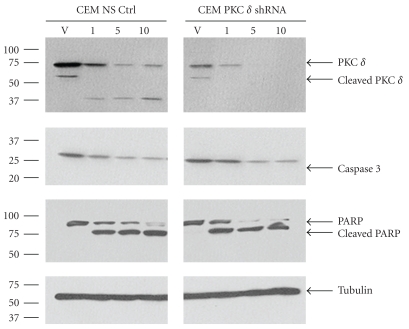
Suppression of PKC *δ* promotes PARP cleavage in CCRF-CEM cells. Western blot analysis was performed using antibody against PKC *δ*, Caspase 3, PARP, and Tubulin on total lysate (0.25 × 10^6^ cell equivalents) from CCRF-CEM transfectant cells with control shRNA or CCRF-CEM transfectant cells with PKC *δ* shRNA that were treated with vehicle (lane marked V; 0.1% DMSO), 1 *μ*M fenretinide (lane marked 1), 5 *μ*M fenretinide (lane marked 5), or 10 *μ*M fenretinide (lane marked 10) for 24 hours.

**Figure 14:**
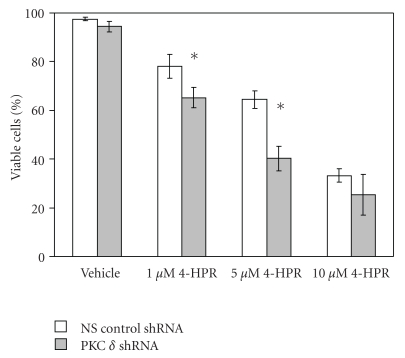
Suppression of PKC *δ* promotes fenretinide-induced cell death in CCRF-CEM cells. Cell death of CCRF-CEM transfectant cells with control nonspecific (NS) shRNA and CCRF-CEM transfectant cells with PKC *δ* (PKC delta) shRNA treated with vehicle (0.1% DMSO) or fenretinide (4-HPR at 1 *μ*M, 5 *μ*M or 10 *μ*M dose) for 24 hours was examined by trypan blue dye exclusion assay. Error bars represent the mean ± S.D. from three separate experiments. Statistically significant differences from cell viability in untreated cells (standard *t*-test; *P* < .05) are marked by “∗”.

**Figure 15:**
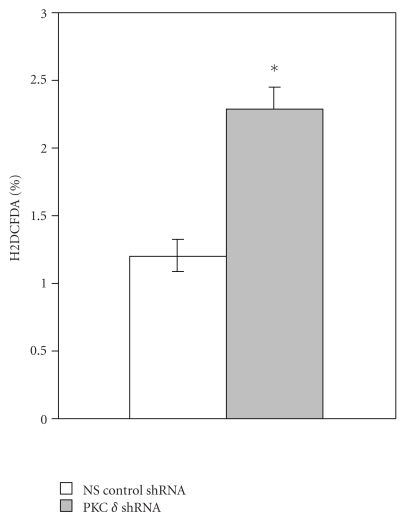
Suppression of PKC *δ* promotes ROS generation in CCRF-CEM. Generation of ROS in CCRF-CEM transfectant cells with control nonspecific (NS) shRNA and CCRF-CEM transfectant cells with PKC *δ* (PKC delta) shRNA was examined using FACSCAN analysis of Carboxy-H2DCFDA stained cells. Error bars represent the mean ± S.D. from three separate experiments. Statistically significant differences from cell viability in untreated cells (standard *t*-test; *P* < .05) are marked by “∗”.

To determine if cleaved PKC *δ* was required for fenretinide-induced apoptosis, expression of the kinase was suppressed in CCRF-CEM cells by shRNA. The shRNA plasmids were introduced into cells by lentiviral transduction. As shown in Figure 13, shRNA targeting PKC *δ* resulted in significant loss of protein when compared to the cells transduced with the negative control. Control shRNA CCRF-CEM cells and cells expressing PKC *δ* shRNA were treated with vehicle (0.1% DMSO) or with 1 *μ*M, 5 *μ*M, or 10 *μ*M fenretinide for 24 hours and cell death was measured by trypan blue dye exclusion. As shown in Figure 14, CCRF-CEM cells expressing PKC *δ* shRNA were more sensitive to fenretinide compared to control (i.e., ~60% cell death versus ~36% cell death with 5 *μ*M drug, resp.). The increase in fenretinide-induced death in the CCRF-CEM cells expressing PKC *δ* shRNA compared to control cells was significant at drug concentrations of 1 *μ*M and 5 *μ*M (i.e., *P *= .03 and *P* = .002, resp.). As shown in Figure 13, CCRF-CEM cells expressing PKC *δ* shRNA displayed more complete PARP cleavage with higher doses of fenretinide compared to control cells though there was not an observed increase in Caspase 3 cleavage. CCRF-CEM cells expressing PKC *δ* shRNA displayed a complete loss of both full length and cleaved PKC *δ* when treated with fenretinide (Figure 13). Suppression of PKC *δ* did lead to an increase in basal ROS levels in CCRF-CEM cells. As shown in Figure 15, cells with PKC *δ* shRNA exhibited a nearly 2 fold increase in ROS production compared to control (*P *<.001). The increased sensitivity to fenretinide in the cells with PKC *δ* shRNA is reminiscent of observations with REH cells treated with a combination of Bryostatin-1 and fenretinide. Bryostatin-1 in combination with fenretinide resulted in complete loss of PKC *δ* in REH cells (Figure 12) and the PKC agonist promoted sensitivity to fenretinide (Figure 11(a)). These results suggest that the role for PKC *δ* in fenretinide-induced cell death in the leukemia cells is complex. 

## 4. Discussion

The findings of this study suggest that PKC *δ* may play an important role in fenretinide-induced apoptosis in lymphoid leukemia cells. Previous studies have established that the synthetic Vitamin A analog acts via diverse mechanisms but a role for any particular PKC isoform has remained elusive [1–6]. An understanding of how fenretinide kills malignant cells is critical since the drug has promise as both a chemotherapeutic agent and a chemoprevention agent [1, 2, 43]. Fenretinide is well tolerated by patients and has shown promise for treating children with neuroblastoma [43–45]. The effectiveness of fenretinide in killing ALL derived cells while sparing nonmalignant lymphoid cells suggests that it may be an effective agent in the therapy of ALL, especially as it is so well tolerated in children [5, 9, 10, 18, 45]. Furthermore, the ability of fenretinide to overcome ABT-737 resistance in ALL cell lines suggests that fenretinide may be useful in combination with BH3 mimetic drugs like ABT-737 [18].

A role for PKC *δ* in fenretinide-induced apoptosis in the leukemia cell lines seemed logical as many of the reported effects of the drug would be expected to impact PKC *δ* mediated signaling (e.g., stimulation of ROS production, stimulation of sphingolipid production, activation of JNK) [6, 8–10]. In the leukemia cells treated with fenretinide, there was a positive correlation between drug-induced apoptosis, drug-induced ROS production, and drug-induced PKC *δ* cleavage (Figures 1, 2, 6, and 7). Fenretinide was shown to promote nuclear translocation of PKC *δ* (Figure 8) which is an important event in apoptotic signaling involving the kinase [33–36]. The antioxidant Vitamin C protected all the leukemia cells from fenretinide-induced cell death (Figure 3) and prevented cleavage of the kinase (Figure 10). These findings would suggest that ROS might promote PKC *δ* cleavage to initiate proapoptotic signaling. While this notion might prove true, a recent study has suggested that PKC *δ* regulates ROS production as primary fibroblasts from PKC *δ* knockout mice or mice expressing a dominant negative mutant of PKC *δ* exhibit increased production of ROS in response to UV irradiation [46]. Consistent with such a notion, the suppression of PKC *δ* by shRNA in CCRF-CEM cells promoted basal ROS production (Figure 15) and sensitized the cells to fenretinide-induced apoptosis (Figure 14). The findings that inhibition of ROS blocks PKC *δ* cleavage (thus suppressing activation of prostress signaling mediated by the kinase) while loss of PKC *δ* enhances ROS production suggest that PKC *δ* may regulate ROS and in turn, ROS may regulate the kinase. Such a mechanism is not too different from the relationship between PKC *δ* and Caspase 3; the protease cleaves and activates PKC *δ* but the kinase in turn activates Caspase 3 [33–36].

The production of sphingolipids has been shown to be an important event in fenretinide-induced apoptosis [1, 3, 9, 10]. PKC *δ* on the one hand has been shown to be activated by the sphingolipid ceramide, and on the other hand, promotes ceramide production [47, 48]. In response to etoposide, PKC *δ* is translocated to the mitochondria and promotes ceramide production in prostate cancer cells [48]. The Maurer group recently demonstrated that fenretinide promotes production of dihydroceramide rather than ceramide [10]. Interestingly, whereas Bryostatin-1 protects REH cells from etoposide [27], the PKC agonist promotes cell death in response to fenretinide (Figure 11(a)). While it was shown that activation of PKC *α* appears to be critical in protection of the REH cells from etoposide [27], Bryostatin-1 suppressed PKC *δ* expression (Figure 12), and potentiated fenretinide-induced cell death in the REH cells (Figure 11(a)). Perhaps the differences may be due to different effects of ceramide and dihydroceramide on PKC *δ* mediated signaling pathways and for that matter pathways regulated by other PKC isoforms. However, a role for dihydroceramide as a regulator of cell death is only just emerging, so much work is needed to establish how dihydroceramide might promote cell death. It will be helpful to understand how PKC *δ* may regulate and be regulated by dihydroceramide. 

PKC *δ* has been implicated as a positive regulator of JNK, particularly during stress signaling leading to apoptosis [49]. Activation of JNK in CCRF-CEM cells leading to MCL-1 degradation observed by Kang and colleagues [18] could be the result of PKC *δ* activation of JNK. However, the loss of MCL-1 observed in RS4;11 cells in the present study occurs in the apparent absence of JNK activation (Figure 6). The correlation between fenretinide-induced PKC *δ* cleavage and loss of MCL-1 in the drug sensitive RS4;11 and CCRF-CEM cells (Figures 6 and 7) is consistent with reports demonstrating that PKC *δ* cleavage product phosphorylates MCL-1 resulting in its proteasomal degradation [38]. However, fenretinide-induced degradation of MCL-1 is not required for drug-induced apoptosis since REH cells actually express increasingly higher amounts of the antiapoptotic protein with higher concentrations of fenretinide (Figure 6) but high concentrations of the drug are still toxic to the cells (Figure 1). Fenretinide (10 *μ*M) does promote MCL-1 gene expression after 6 hours in REH cells (Figure 9), so the increased expression of MCL-1 protein may reflect an increase in gene expression. The cleaved PKC *δ* produced in the REH cells may not be sufficient to promote degradation of MCL-1. These findings would suggest that PKC *δ* may not be as important in fenretinide-induced apoptosis in the REH cells. Still, the finding that Bryostatin-1 potently suppresses PKC *δ* expression and potentiates fenretinide-induced cell death suggests that PKC *δ* would suggest otherwise. Identification of PKC *δ* targets other than MCL-1 will provide a better understanding of how the kinase regulates fenretinide-induced apoptosis. One possible mechanism may involve regulation of ROS production. While we may not fully understand how PKC *δ* regulates fenretinide-mediated apoptosis in lymphoid leukemia cells, it is clear that the kinase may be a useful target for optimizing antileukemia strategies that make use of fenretinide.
